# Large-Scale Production of Nanographite by Tube-Shear Exfoliation in Water

**DOI:** 10.1371/journal.pone.0154686

**Published:** 2016-04-29

**Authors:** Nicklas Blomquist, Ann-Christine Engström, Magnus Hummelgård, Britta Andres, Sven Forsberg, Håkan Olin

**Affiliations:** 1 Department of Natural Sciences, Mid Sweden University, SE-851 70 Sundsvall, Sweden; 2 STT Emtec AB, SE-852 29 Sundsvall, Sweden; Institute for Materials Science, GERMANY

## Abstract

The number of applications based on graphene, few-layer graphene, and nanographite is rapidly increasing. A large-scale process for production of these materials is critically needed to achieve cost-effective commercial products. Here, we present a novel process to mechanically exfoliate industrial quantities of nanographite from graphite in an aqueous environment with low energy consumption and at controlled shear conditions. This process, based on hydrodynamic tube shearing, produced nanometer-thick and micrometer-wide flakes of nanographite with a production rate exceeding 500 gh^-1^ with an energy consumption about 10 Whg^-1^. In addition, to facilitate large-area coating, we show that the nanographite can be mixed with nanofibrillated cellulose in the process to form highly conductive, robust and environmentally friendly composites. This composite has a sheet resistance below 1.75 Ω/*sq* and an electrical resistivity of 1.39×10^-4^ Ωm and may find use in several applications, from supercapacitors and batteries to printed electronics and solar cells. A batch of 100 liter was processed in less than 4 hours. The design of the process allow scaling to even larger volumes and the low energy consumption indicates a low-cost process.

## Introduction

A cost-efficient and large-scale process of highly conductive carbon nanoparticles, such as graphene and nanographite, is essential to take the step from laboratory experiments to useful commercial products [1, 2]. Carbon nanoparticles and composites will be required for various applications, such as supercapacitors [3, 4], batteries [4, 5], printed electronics [6], or solar cells [4, 5]. These applications will need a cost-effective exfoliation process and preferably environmentally compatible solvents where the ideal is aqueous processing.

Exfoliated graphite comes in different qualities [7] from single-layer, bi-layer, few-layer (2–5 layers), multilayer graphene, and graphite nanosheets. Here, we define nanographite as a mix of all these qualities, with a certain size distribution. The requirement for electrodes, for supercapacitor and other applications, is demanding and rather different from applications that require only few-layer graphene. In these electrode applications the combined large-area and high conductivity as well as ability to coat are the most important ones. Most reports about graphite exfoliation processes aim to produce only graphene or few-layer graphene, and the partially exfoliated material are not used. This makes it hard to compare parameters like size distribution and production rate of various exfoliation processes designed for different uses and applications.

There is still no good method to precisely determine the yield, and thereby the production rate, in large-scale of a certain quality of exfoliated graphite, even if progress is emerging [8–12]. To avoid the time-consuming work of identify, specify and count every particle in the processed batch, UV-VIS spectroscopy is widely used to measure concentration together with TEM, AFM, Raman spectroscopy etc. to determine the particle quality [9–12]. It is worth noting that measurements on graphene and graphene-like particles are difficult, due to their propensity to agglomerate together and form few-layer, multi-layer, and finally graphite [12]. Therefor, to be able to compare the production rate of similar material, we need to estimate the ratio of few-layer produced in our process. This despite the fact that we, in our application, use the produced nanographite as is.

To exfoliate graphene from graphite in solution, sonication is the standard laboratory procedure. However, this method is difficult to scale due to that the concentration scales roughly inversely with liquid volume and the process is not energy efficient [9, 13, 14]. The extended treatment time results in a low throughput and the graphene sheets might be cut to smaller flakes during the exfoliation process. Other approaches to wet exfoliation are jet cavitation [15], ball milling [16], rotational dispersers [10, 17], wet grinding [18], and homogenizer processing [11]. These methods are potential candidates for large-scale production, however, as Paton et al. point out [10], the production rates of most of these methods is less than 0.4 gh^-1^. To demonstrate large-scale exfoliation, Paton et al. [10] show that large quantities of defect free graphene can be made in N-methyl-2-pyrrolidone (NMP) suspension by high-shear rotational mixing of graphite. They show a few-layer graphene production rate of 5.3 gh^-1^ and estimate that it could be scaled up to a production rate exceeding 100 gh^-1^ for a 10 m^3^ process scale. Nacken et al. [11] used a commercial high-pressure homogenizer to prove large-scale exfoliation of graphite in NMP and water-surfactant mixtures, with a production rate of 0.5 gh^-1^ of few-layer graphene.

In addition to a process with high throughput of conducting carbon nanoparticles, when making thin films and electrodes, the coating formulation will be of importance as well as adhesion properties. Therefore, the ability to form composites of the processed material will be essential. Graphene or nanographite composites have many applications for example in energy storage and harvesting [4] to phase change materials [19]. Malho et al. [2] have shown, by sonication, that graphene can be exfoliated directly from graphite in aqueous environment with nanofibrillated cellulose (NFC) as the only dispersant, forming a stiff, tough and strong nanocomposite. NFC can be used as binder in nanographite electrodes for supercapacitors to improve electrical and mechanical properties [3].

The combination of these problems point to the need to develop a process that is preferably water based, is suitable for large-scale production, and permit incorporation of binders to enable further processing. The process should also be energy-efficient, have high yield and meet the requirements of the specific application. For example, an electrode application such as supercapacitors require a certain size distribution and ability to add other carbon qualities, such as activated carbon, leading to high electrical conductivity combined with high active surface area.

In this paper we describe a novel type of process for large-scale exfoliation in an aqueous environment. We study the particle size distribution and the degree of delamination during the process. We also analyzed the sheet resistance and surface area of the nanographite-NFC composite to compare the material with other conducting carbon alternatives. The majority of the particles in the nanographite was nanometer-thin and micrometer-wide flakes with a graphene-like structure. The production rate was 500 gh^-1^ of nanographite.

## Materials and Methods

To prepare the suspension for exfoliation, 20 gL^-1^ thermally expanded graphite (SO#5-44-04) from Superior Graphite was mixed in water with an addition of 2 wt% polyacrylic acid, in relation to the graphite amount, as dispersant. The suspension volume was 100 L. The pH was adjusted to 3.5 by sulfuric acid in order to get a fixed and reproducible pH-value of the suspension during exfoliation. The expanded graphite has initially low pH when mixed with water, due to residual acid from the manufacturing process.

To exfoliate, the suspension was forced by a high-pressure pump through a 1 m long tube with an inner diameter of 2 mm. The pressure was held constant at 50 bar with an initial flow rate of 4.95 L min^-1^, giving rise to a shear rate of 1×10^5^ s^-1^. This procedure was repeated 10 times (10 passes). The dynamic viscosity increased slightly for each passing leading to a decreased flow rate at constant pressure. The flow rate decreased from 4.95 L min^-1^ to 4.27 L min^-1^ during the 10 passes, which corresponds to a change in dynamic viscosity from 23.7 mPas to 27.6 mPas. The calculated shear rate went from 1.05×10^5^ s^-1^ down to 0.91×10^5^ s^-1^. The calculated Reynolds number (Re) decreased from initially 2217 to 1642. To calculate the shear rate, $\dot{\gamma }$, for laminar flow in a straight tube we used [20]

$$\begin{array}{c} \dot{\gamma }=\frac{4Q}{\pi r^{3}}, \end{array}$$ (1)

where *Q* is the volumetric flow rate, and *r* is the hydraulic pipe radius. To calculate the Reynolds number we used [20]

$$\begin{array}{c} Re=\frac{2Q\rho }{\mu \pi r}, \end{array}$$ (2)

where *ρ* is the fluid density and *μ* is the dynamic viscosity of the fluid. The production rate, *P*_R_, can be expressed by [14]

$$\begin{array}{c} P_{R}=\frac{CV}{t}, \end{array}$$ (3)

where *C* is the concentration or solid content of processed material, *V* is the liquid volume (batch volume), and *t* is the production time.

**Fig 1:**
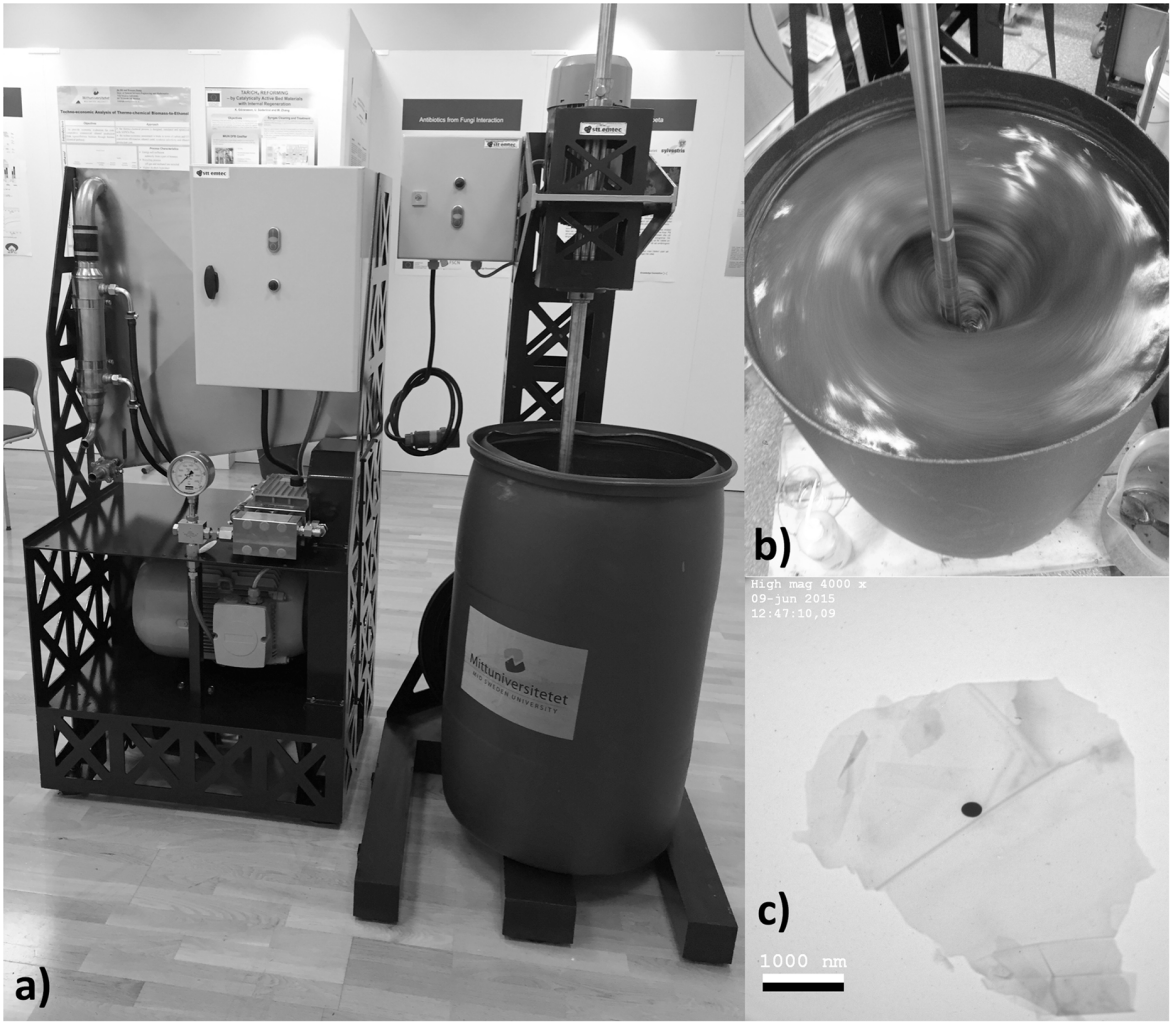
The exfoliation equipment. a) The hydrodynamic tube shearing device with dispersion barrel and stirrer, b) the dispersion barrel during stirring (to avoid graphite flotation) and, c) a TEM-image of the processed material.

Samples of the suspension were taken during the process at 0, 5 and 10 passes to examine the change in particle structure. After 10 passes the pH was adjusted to 7 with sodium hydroxide and the suspension was divided in two batches, A and B. Fig 1 shows the exfoliation equipment.

To get robust freestanding films for measurements from the suspension, NFC was added as binder. The NFC used was TEMPO-oxidized kraft-pulp NFC made using the method described in [21]. In order to examine the addition of NFC for improved composite properties two different methods were used.

In batch A, 10 wt% NFC in relation to the graphite amount were added to the suspension during gentle stirring for 40 min. The suspension was then dispersed in two further passes in the tube shearing process with the parameters stated above in order to exfoliate the NFC and get a well-dispersed composite. In batch B, NFC was added in the same way but processed by high speed shearing with an IKA T25 digital Ultra Turrax disperser instead. 1 liter suspension was exfoliated with dispersing element S25N-10G in the Ultra Turrax at 12 000 rpm for 10 min.

### Sample preparation and analysis

To analyze the structural change in the material, sample grids for a transmission electron microscope (TEM) were prepared. Suspensions from 0, 5 and 10 passes were diluted to 0.0047% solids content and one droplet (30 mg) was applied to the center of a TEM-grid. Five grids per dispersion were prepared. The microscope used was a JEOL-2000FX.

To analyze the particle size distribution in the suspensions from 5 and 10 passes, one droplet of the diluted suspensions (0.0047%) was applied on an aluminum scanning electron microscope sample stub. The particles were characterized in size by image analysis in a ZEISS EVO-50 SEM. In order to determine the particle size, predefined squares in the image were used where each particle could fit in. A total of 2645 particles were characterized from the two suspensions. To estimate the concentration of the smallest particles, UV-VIS spectroscopy was performed on the top phase of a sedimented sample from 10 passes with a Shimadzu 1800 spectrophotometer (absorbance at 660 nm with an extinction coefficient of 1060 mL mg^-1^m^-1^).

To analyze the particle thickness in the processed material, samples for Atomic Force microscopy (AFM) were prepared. Suspension from 10 passes were diluted to 0.0047% solids content and one droplet (30 mg) was applied to the center of 1×1 cm silicon wafer substrate. Samples from the top phase of a sedimented 10 pass suspension was also prepared. Four samples per dispersion were prepared. The microscope used was a Dimension AFM with a Nanoscope IIIa controller (Digital Instruments).

Composite films were made by filtering batch A and B respectively on Millapore Durapore Membrane Filter (filter type: 0.22 μm GV) in four different quantities, 0.1 g, 0.25 g, 0.5 g and 1 g, dry weight. Films of the unexfoliated dispersion (expanded graphite, water and polyacrylic acid) were also prepared in the same way, but due to its powdery unstable structure, no measurements could be done on these samples.

The sheet resistance of the films was measured using a four-point probe Keithley 2611A system after 24 h in room temperature. To analyze the surface area of the composite we used the Brunauer–Emmett–Teller (BET) method. The BET-samples were made from suspensions with 0 passes, batch A and B by instantly freezing the suspensions with liquid nitrogen followed by freeze-drying to form a dry powder. The BET measurements were made by Vesta Lab Sweden AB.

## Results

### Particle structure analysis

**Fig 2:**
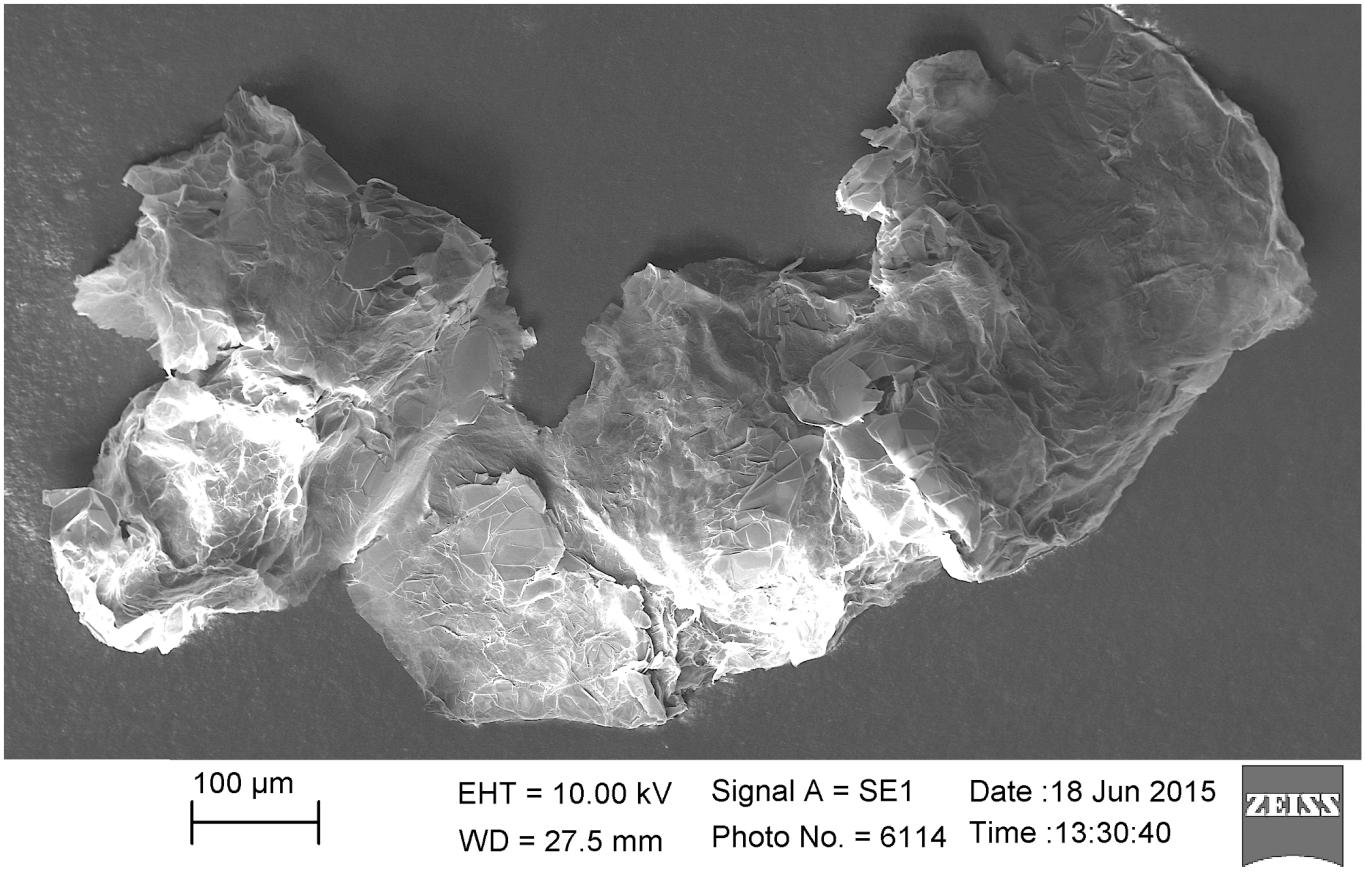
Initial material. SEM-image of the initial graphite, before exfoliation (0 passes).

Fig 2 shows the structure of the initial material. The thermally expanded graphite consists of large granulates, several hundred micrometers in size. These granulates have a partly cracked surface that looks like tightly packed graphite flakes.

**Fig 3:**
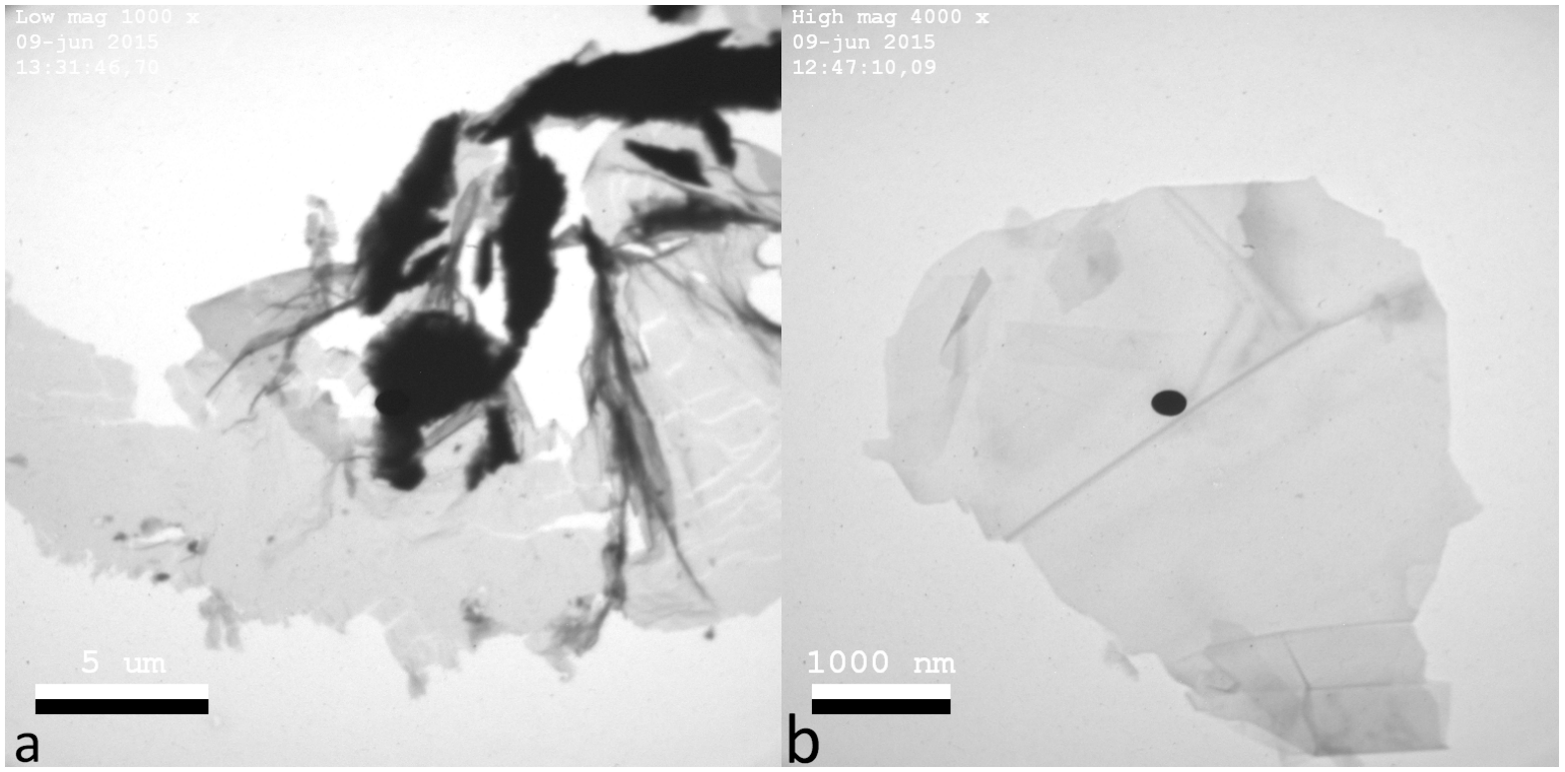
TEM-imaging. TEM-images of particles from the process. a) after 5 passes, a typical particle found in the suspension that fits the frame size 20×20 μm. The scale is 5 μm. b) after 10 passes, a typical particle that fits the frame size 2.5×2.5 μm. The scale is 1000 nm.

Fig 3a shows a typical particle found in the suspension after 5 passes and Fig 3b is a typical particle found in the suspension after 10 passes. It can be seen in 3a that the material is partly exfoliated and has some cracks. After further exfoliation (10 passes), we can find a larger amount of well-exfoliated flakes that look like graphene, see Fig 3b. Note that the scale is different in the two images. Thin flakes can be found in both suspensions but we can clearly see that the amount of well-exfoliated flakes increases with increased amount of passes. The flakes are significantly large, and their transparency suggests that we obtained one or few layer graphene flakes. More TEM-images are found under section A in S1 File.

**Fig 4:**
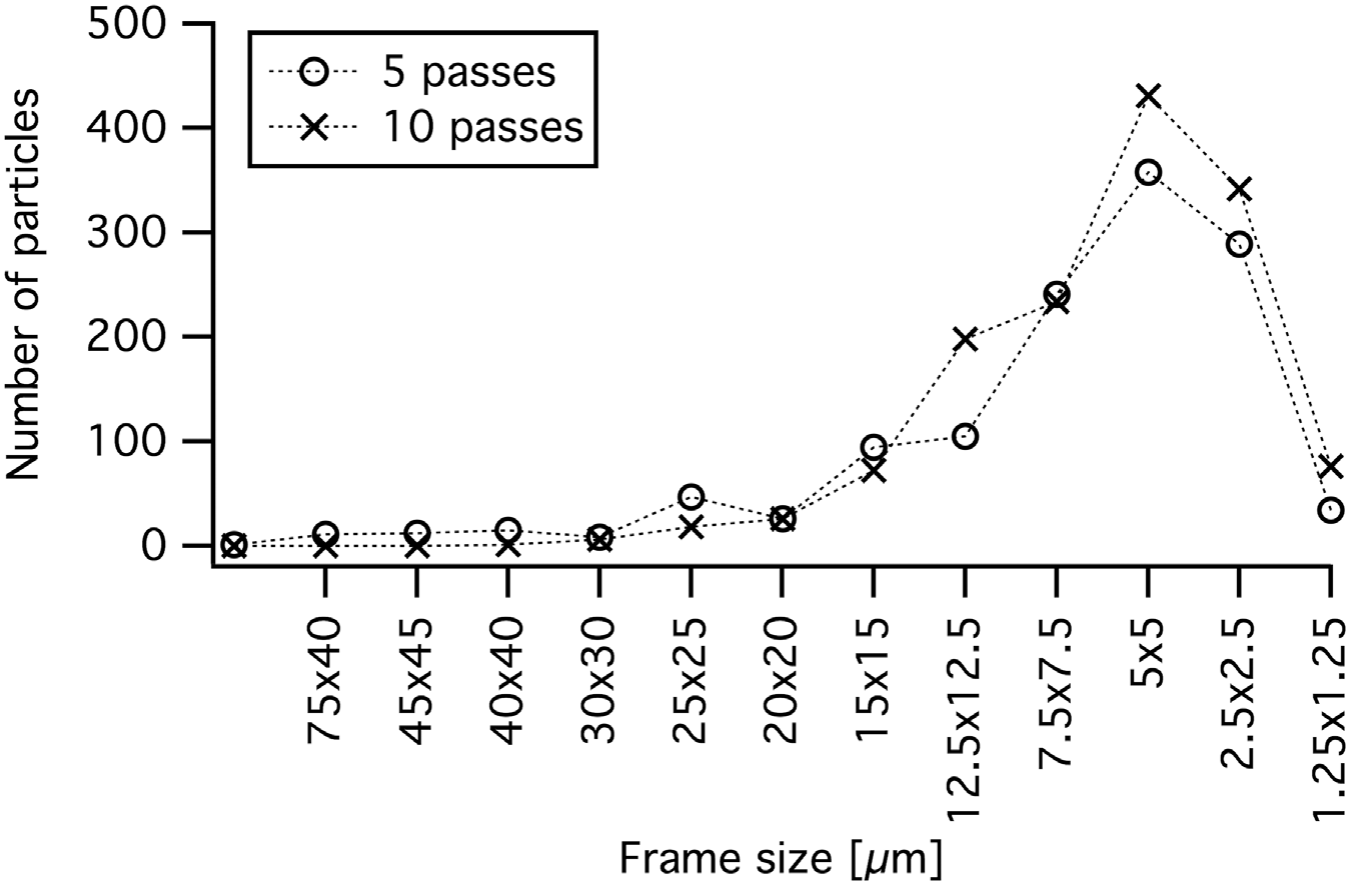
Particle size distribution. Particle size distribution from SEM image analysis.

Fig 4 shows the particle size distribution from the SEM image analysis. It can clearly be seen on the SEM images, that the suspension from 10 passes has much fewer large and thick particles than the 5 pass sample and a larger amount of small thinner flakes. The frame size represents the surface size of the particles and does not state the particle thickness. The particles transparency in the SEM analysis was used to determine if they were thick or thin. The particles appear to become thinner with increasing number of passes, but retain a relatively high surface area. A selection of SEM images are found under section B in S1 File. In TEM even smaller thin flakes, in the order of a few hundred nanometers, can be found. These were difficult to observe in the SEM due to the instrumental resolution, so the amount of flakes with surface size below 1 micrometer was probably much higher than the stated value in Fig 4. UV-VIS spectroscopy indicates that these fractions, frame size 2.5×2.5 μm^2^ and smaller, corresponds to roughly 4% of the processed batch.

**Fig 5:**
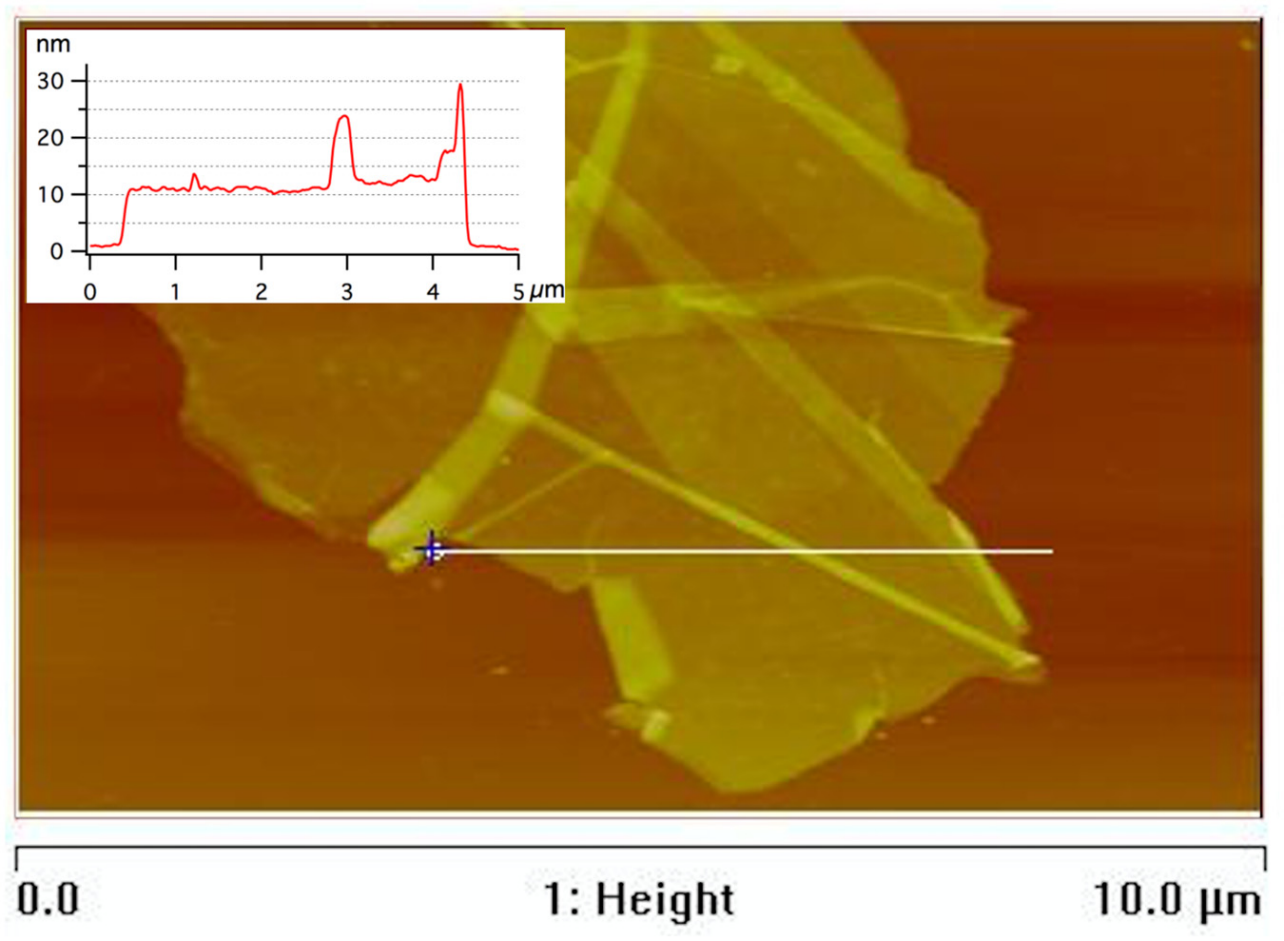
Thickness measurement. AFM image of a partly folded flake found in the suspension after 10 passes with corresponding height profile along the indication bar. The measured flake thickness was 9.12 nm and the flake fits frame size 7.5×7.5 μm.

Fig 5 shows a AFM-image of a typical particle found in the suspension after 10 passes, with the corresponding height profile. It can be seen from the AFM measurements that most of the nanographite flakes are partly folded or wrinkled and not flat against the silicon wafer substrate. The measured average flake thickness after 10 passes was in the range of 10 nm to 20 nm. More AFM images can be found in Section C in S1 File.

### Electrical properties

**Fig 6:**
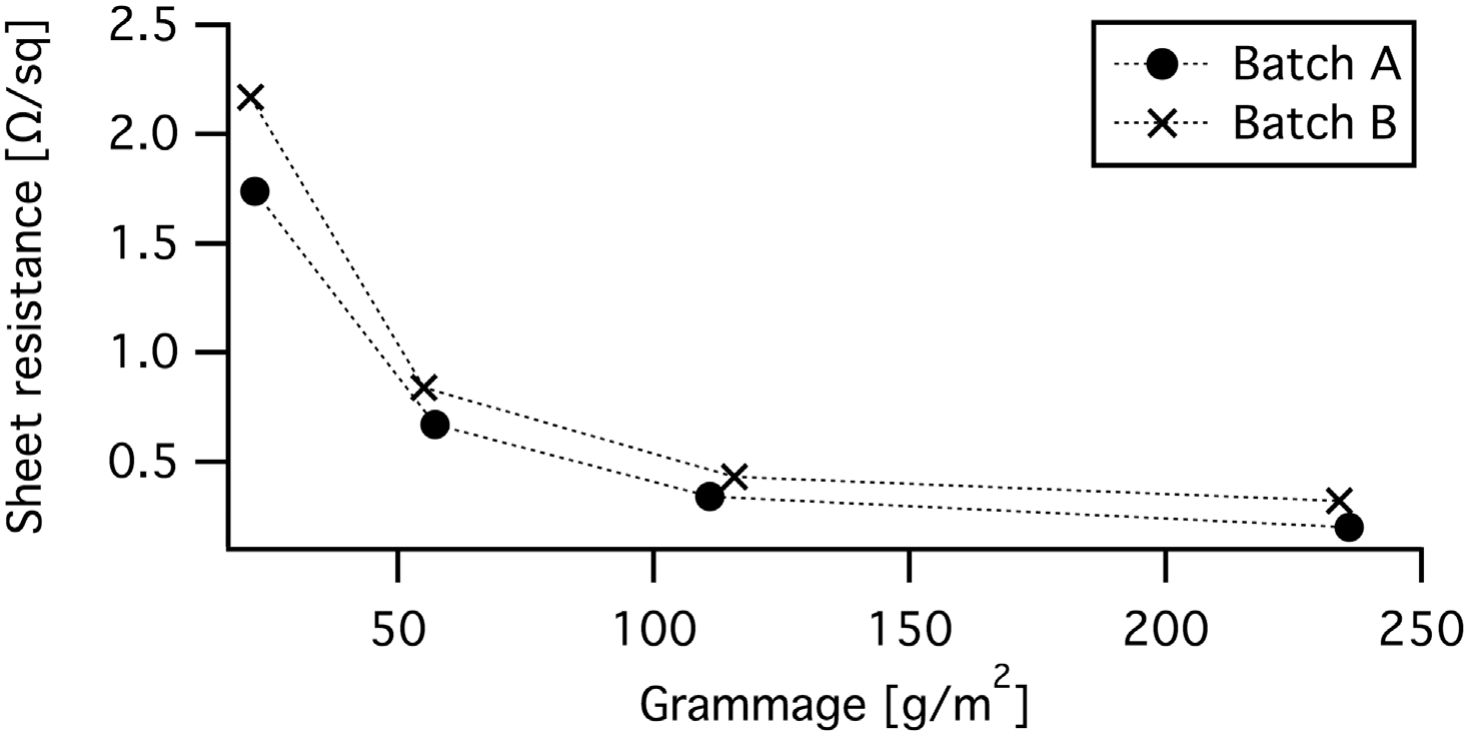
Sheet resistance. Sheet resistance *Rs* of composite films from batch A and B. The grammage was calculated from the weight and area of the each composite film. The standard deviation was too small to be visualized in the figure.

Sheet resistance was measured on each film from batch A and B. As shown in Fig 6, the sheet resistance decreased with increased grammage and batch A had an overall lower sheet resistance than batch B. The standard deviation was between 0.01 to 0.03 Ω/*sq* for batch A and 0.01 to 0.12 Ω/*sq* for batch B. The biggest deviation was measured on the films with the lowest grammage.

To compare the composite to commercial highly conductive carbon alternatives, the electrical resistivity was calculated by multiplying the sheet resistance with the film thickness. The electrical resistivity of Batch A was 1.39×10^-4^ Ωm, with a standard deviation of 0.25×10^-4^ Ωm. Batch A had an electrical resistivity of 1.21×10^-4^ Ωm, with a standard deviation of 0.39×10^-4^ Ωm. The highly conductive carbon alternatives used for comparison was battery graphite, carbon black and carbon ink. This types of carbon are often used to enhance the electric conductivity in electrodes and conducting layers. Comparative data was retrieved from the manufacturer and from published papers. The battery graphite was also mixed with NFC, in the same way as batch B, to see how the binder affects the electric properties. The comparison is presented in Table 1. The BET data is only added to be able to compare the resistivity to other carbon alternatives, since a very porous structure is expected to have a higher resistivity than a compact one.

**Table 1:** Comparison of electrical resistivity and BET surface area for alternative conducting carbons. *ρ* is the electrical resistivity, SSA is the specific surface area of the material, CB is carbon black and BG is battery graphite.

Sample	Binder	*ρ* [Ωm]	SSA [m^2^g^-1^]
*BatchA*	10% NFC	1.39⋅10^-4^	21.0
*BatchB*	10% NFC	1.21⋅10^-4^	22.4
*BG*, *ABG*2025^a^	10% NFC	8.09⋅10^-4^	not specified
*BG*, *ABG*2025^a^^,^^b^	compressed	6.61⋅10^-4^	19.8
*CB*, *VulcanXC*—72 [22]	compressed	1.35⋅10^-3^	254.0
*CB*, *GPN*991 [22]	compressed	1.12⋅10^-2^	9.8
*LoctiteScreenprint—carbonink* ^b^	not specified	3.56⋅10^-4^	not specified

Table notes

^a^ Ref. [3]

^b^ According to the manufacturer.

## Discussion

### Production rate and scalability

To process a batch of 100 L nanographite with a solids concentration of 20 gL^-1^ under the described shear conditions, with a mean flow (*Q_mean_*) of 4.6 Lmin^-1^, took roughly 4 h and consumed about 20 kWh of electricity. This resulted in a production rate (*P_R_*) of 500 gh^-1^. To scale up the production we have to increase the amount of material passing trough the process without changing the shear conditions. Parameters that can be adjusted in the process are flow, graphite concentration, tube diameter, tube length and number of tubes. The concentration affects the dynamic viscosity and thus both pressure and Reynolds number. The tube diameter directly affects the shear rate and the tube length affects the pressure and shear time. To scale the process without any affect of the shear conditions we can adjust the flow according to the number of shear zones (tubes), *N_tube_*. This can be expressed by

$$\begin{array}{c} P_{R}=500gh^{-1}\cdot N_{tube} \end{array}$$ (4)

where the flow need to be adjusted as *Q_mean_* = *N_tube_* ⋅ 4.6 Lmin^-1^. If the number of tubes, in parallel, doubles together with the flow rate, the shear conditions remains the same but the production rate is twice as high. Thus, scaling is achieved by simply increasing the number of tubes, while keeping the shear condition constant.

### Comparison with other exfoliation methods

The exfoliation process, described above, enables a fully controlled production where all of the material in the suspension passes the shear zone one time per pass, and all parameters can be monitored and controlled. The parameters, especially pressure, flow rate and number of passes, used in the process are important for the quality of the output. During the optimization of the process, we saw that high pressures and turbulent flow (Reynolds numbers above 4000) together with numerous passes caused over-shearing and cracked the flakes into smaller fragments. We observed that the geometry of the shear zone was important for the quality of the nanographite; a long straight tube and laminar flow seemed to give rise to sufficient shear forces to exfoliate graphite to large sheets and minimize pinch force, which is likely the cause of cracking the sheets. Strong turbulent flow and poorly defined shear zones result in forces acting on the particles which are hard to calculate.

In contrast, to our well defined shear zone in the tube, a commercial homogenizer, which is designed to process food, is not optimal for exfoliation since both shear and pinch forces are present to tear and smash particles into smaller pieces resulting in too small graphene and nanographite flakes. This is not the case in tube-shearing. The same problem, as with homogenizers, may occur in processes with rotary dispersers (high-shear mixing). In these processes it is difficult to know the forces acting on the particles in the inlet and outlet of the direct shear zone. Another uncertainty with rotary dispersers is to determine how many times the particles have passed the shear zone, when exfoliated and unexfoliated graphite are located in the same container.

Two disadvantage with tube-shearing concerns limitation in the maximun concentration and the design restriction on the tube geometry. The limit of concentration is due to the need for pumping the suspension into the tube that have a maximum allowed viscosity. This problem is not necessarily the case for rotary dispenser but a commercial homogenizer faces the same problem. Design restriction on the tube geometry is due to the requirements, firstly, to be in the region of laminar flow to avoid cracking due to pinch forces, secondly, to maximize the shear rate to increase the exfoliation rate. However, as discussed above, scaling is simply achieved by increasing the number of tubes in the process equipment.

### Composites for electrode applications

One significant difference between the composite films was the film density. In batch A, the NFC was added during tubes-shearing while in batch B the NFC was mixed with nanographite after tube-exfoliation, using a high-shear mixer. The density in batch A was the same for all films which indicates a well-dispersed and stable composite. In batch B we could see large variations in density for the four different films. This may indicate that the hydrodynamic tube shearing process is more suitable to get a well-dispersed composite than the high-shear mixing. This leads to superior coating ability of electrodes on large-area substrates with good mechanical and adhesion properties as compared to pure nanographite which leads to cracked films that do not adhere to the substrate, as seen in [3] for a slightly different system. The specific surface area of the composite was low, most likely because the flakes are stacked on each other forming a compact non-porous material.

There are various applications for exfoliated graphite composites. The nanographite composite produced in the described process had a low sheet resistance and low electrical resistivity, which suit applications as a highly conducting matrix around activated carbons in supercapacitor electrodes. In this application a wide size distribution can be beneficial as it can give rise to more pores and increase the surface area. In comparison with carbon black and battery graphite, the nanographite composite has lower electrical resistivity even though it is uncompressed and contains binder. Another suitable application for the composite is as electrical conductor in printed electronics due to its higher conductivity compared with carbon ink, together with a robust and durable structure.

## Conclusion

We have demonstrated a hydrodynamic tube-shearing process suitable for producing large quantities of nanographite. This process produces micrometer-wide and nanometer-thick flakes of nanographite. The exfoliation occurs in an aqueous environment without any toxic chemicals or organic solvents, making it environmentally friendly. The energy consumption in the process is about 10 kWh per kilogram processed graphite, making it low cost. The process is easy to scale up even further, to increase the production rate, by using multiple tubes in parallel. The process is thus environmentally friendly, cost-efficient, and suitable for industrial implementation. We also demonstrated production of highly conductive and robust carbon composites by adding NFC during the process suitable for large-area coating of electrodes.

## Supporting Information

S1 File Microscope images. PDF-file providing additional images from TEM, SEM and AFM imaging.
(PDF)
