# Supplementary material for: Large-Scale Production of Nanographite by Tube-Shear Exfoliation in Water
Source: PLoS One. 2016 Apr 29;11(4):e0154686. doi: 10.1371/journal.pone.0154686 (PMC4851292; doi:10.1371/journal.pone.0154686)
Supplement: S1 File — PDF-file providing additional images from TEM, SEM and AFM imaging. (PDF) [file pone.0154686.s001.pdf]

Supporting information to "Large-scale Production of Nanographite by Tube-shear Exfoliation in Water"

S1-file

Authors: Nicklas Blomquist [1,2,*],Ann-Christine Engström[1], Magnus Hummelgård[1], Britta Andres[1], Sven Forsberg[1], and Håkan Olin[1].

[*] nicklas.blomquist@miun.se
[1] Department of Natural Sciences, Mid Sweden University, SE-851 70 Sundsvall, Sweden
[2] STT Emtec AB, SE-852 29 Sundsvall, Sweden

Content:

A: Electron microscopy of tube-shear exfoliated nanographite 2

B: Flake size measurements 4

C: Flake thickness measurements 6

A: Electron microscopy of tube-shear exfoliated nanographite

The structural change in the material during the process was analyzed by transmission electron microscopy (TEM) in a JEOL-2000FX. Figure A.1 and A.2 shows typical partly exfoliated particles found in the suspension after 5 passes. Figure A.3 to A.8 shows particles from the suspension after 10 passes. These particles are in general thinner and well exfoliated but often folded or wrinkled.

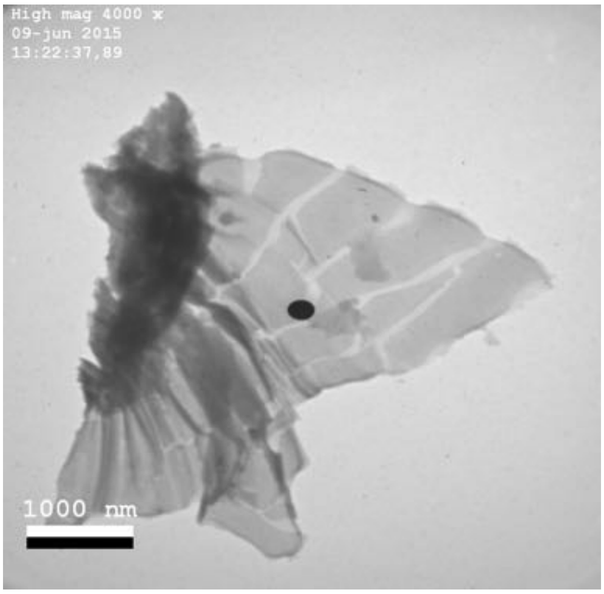

Figure A.1: Partly exfoliated particle found in the suspension after 5 passes.

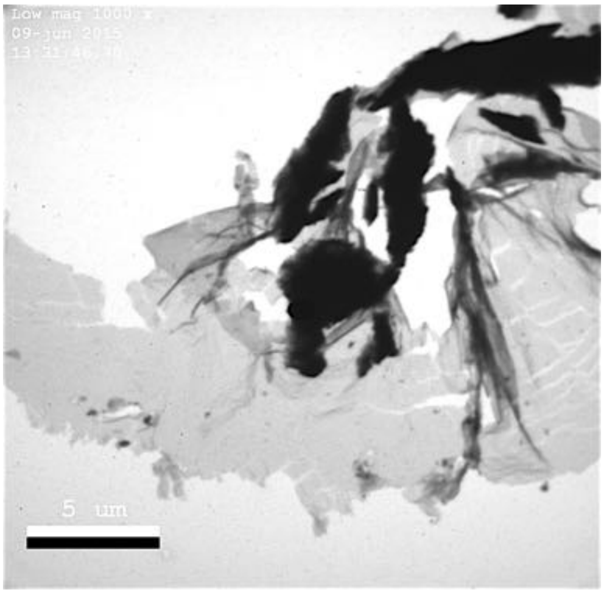

Figure A.2: Partly exfoliated particle with both thick and thin regions, found in the suspension after 5 passes.

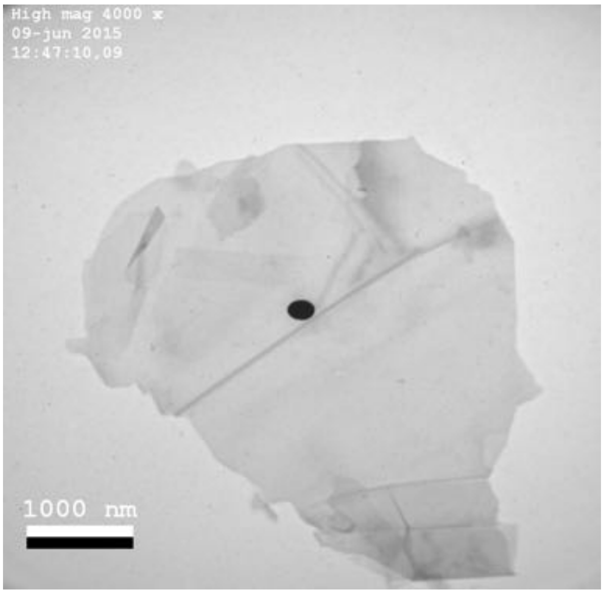

Figure A.3: Exfoliated and partly folded particle found in the suspension after 10 passes.

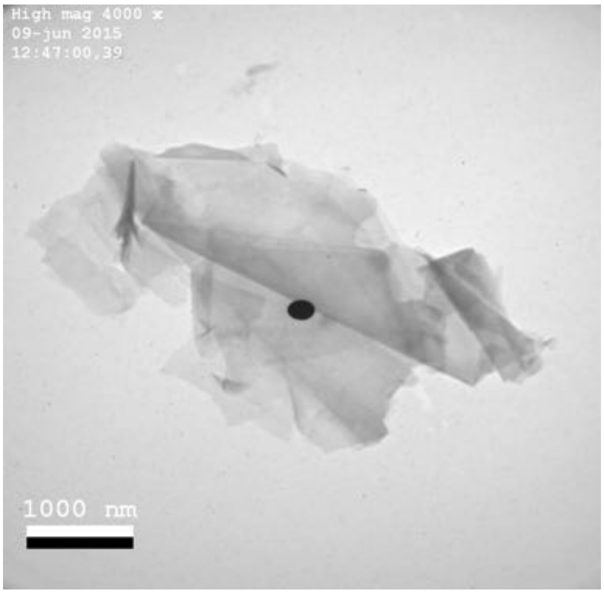

Figure A.4: Exfoliated partly folded and wrinkled particle found in the suspension after 10 passes.

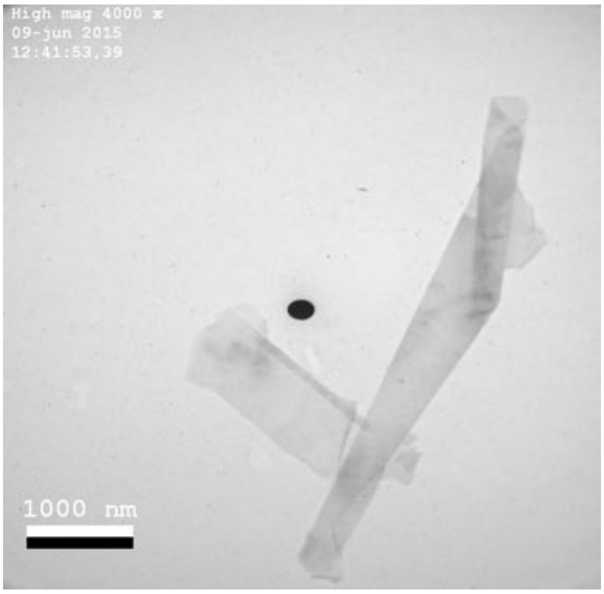

Figure A.5: Small and partly folded exfoliated particles found in the suspension after 10 passes.

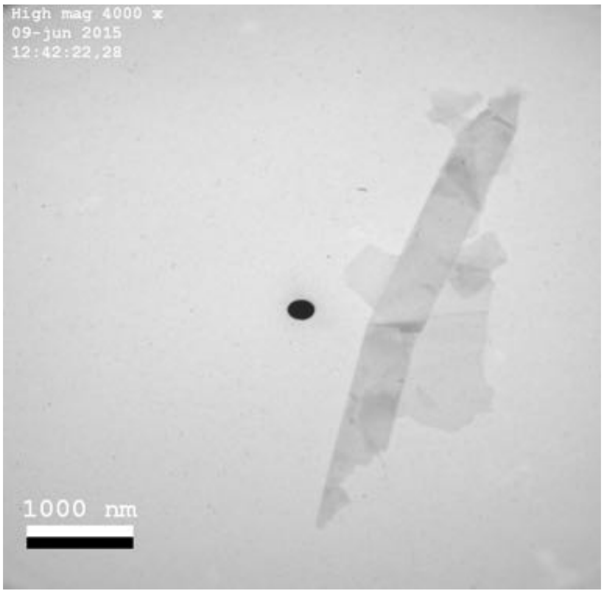

Figure A.6: Small and partly folded and stacked exfoliated particles found in the suspension after 10 passes.

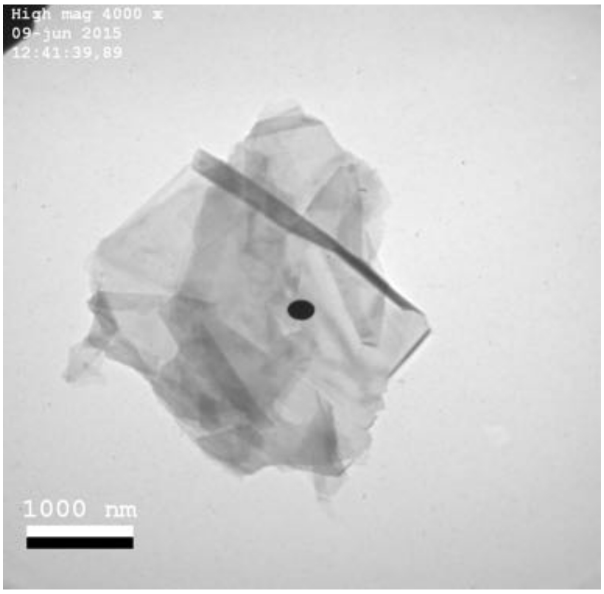

Figure A.7: Folded and partly wrinkled exfoliated particle found in the suspension after 10 passes.

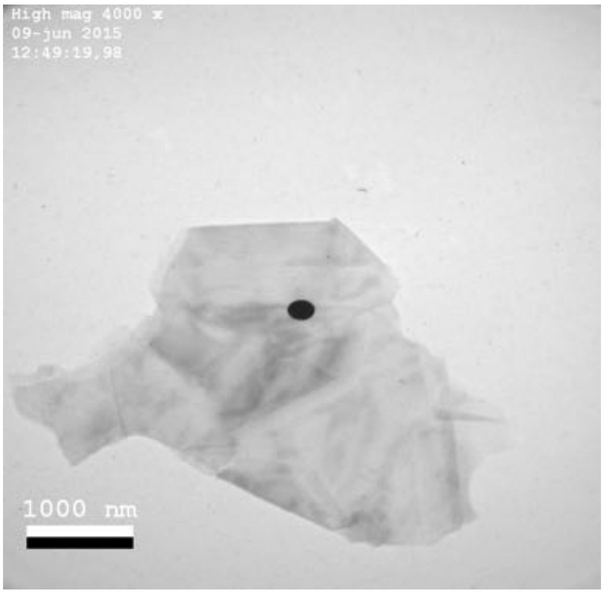

Figure A.8: Wrinkled and partly folded exfoliated particle found in the suspension after 10 passes.

B: Flake size measurements

The particle size distribution of the material was analyzed by image analysis in a ZEISS EVO-50 scanning electron microscope (SEM). Predefined squares were fitted around the particles in the image to determine the particle size. A total of 2645 particles were characterized. A selection of SEM images from the 10pass suspension is showed in figure B.1 to B.3.

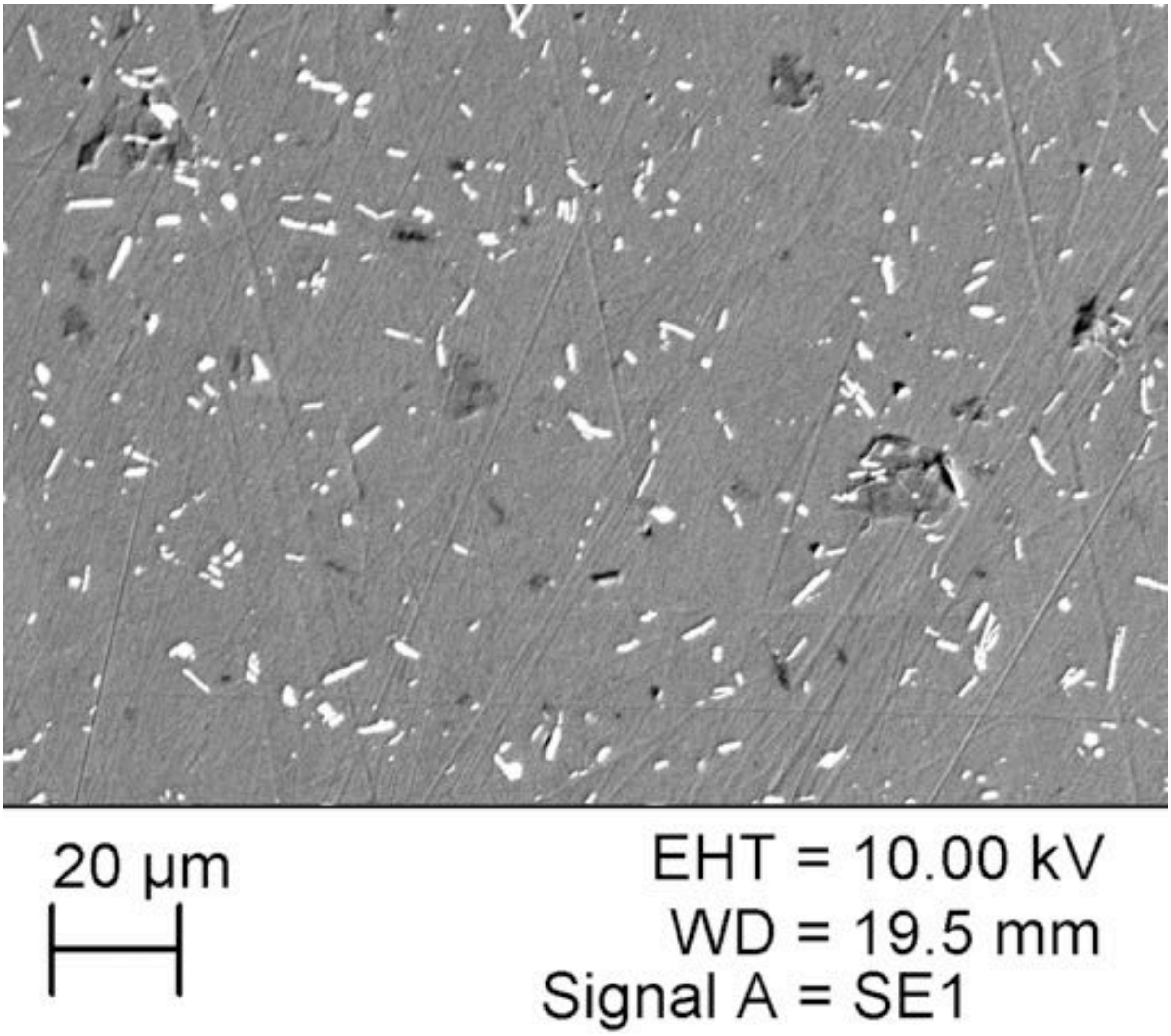

Figure B.1: Enhanced section of an SEM image of the suspension after 10 passes. The thin flakes can be seen as the slightly darker grey in the image and the darker grey represents thicker particles. The white fragments and light grey background corresponds to the aluminum substrate.

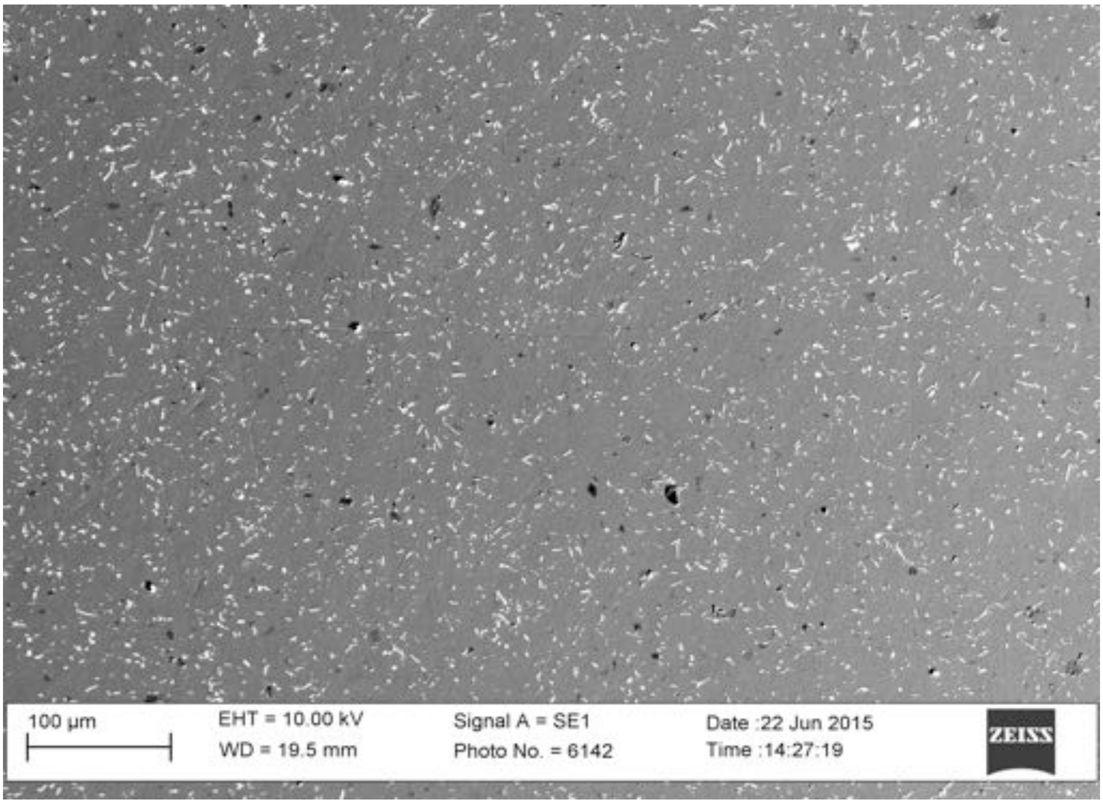

Figure B.2: Image of the suspension after 10 passes. The flakes can be seen as the slightly darker grey to black in the image. The white fragments and light grey background corresponds to the aluminum substrate.

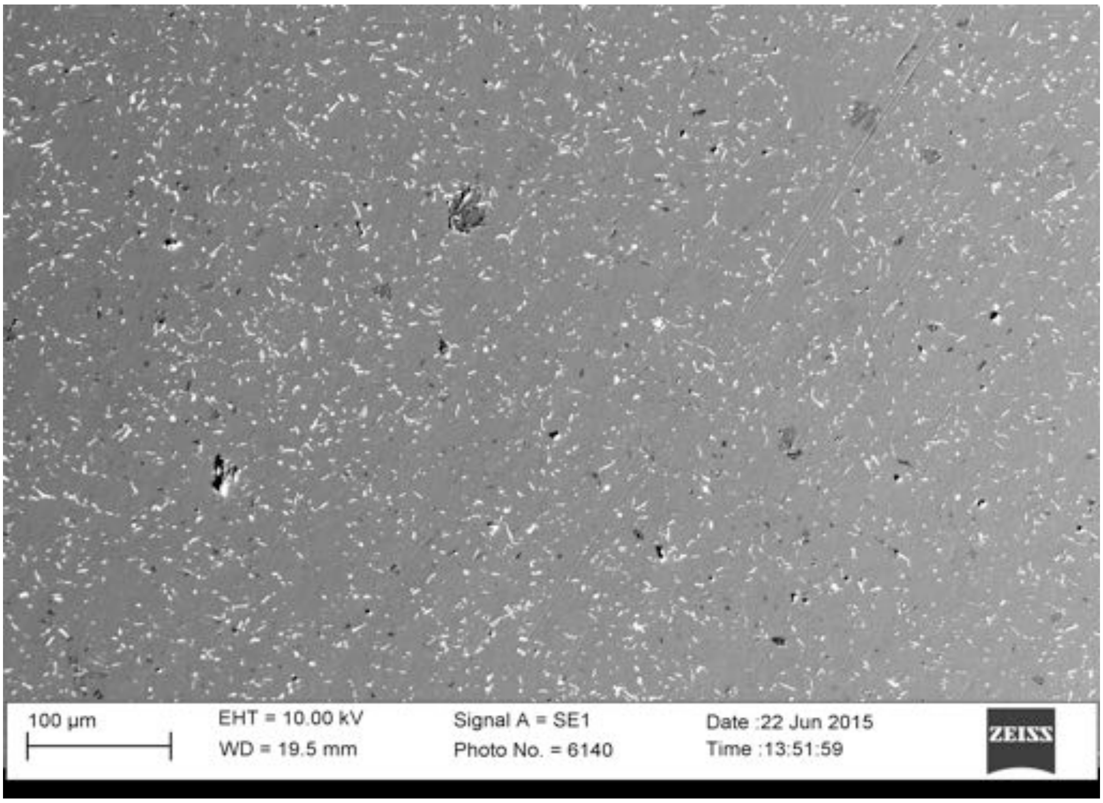

Figure B.3: Another image of the suspension after 10 passes. The flakes can be seen as the slightly darker grey to black in the image. The white fragments and light grey background corresponds to the aluminum substrate.

C: Flake thickness measurements

The flake thickness measurements were carried out by a Dimension atomic force microscope (AFM) with a Nanoscope IIIa controller (Digital Instruments). It can be seen from the AFM measurements that most of the nanographite flakes were partly folded or wrinkled and not flat against the silicon wafer substrate. The height profiles are based on data from the areas on the flakes with minimum wrinkles, indicated with a white line in the topographic height image. The plus sign on the line corresponds to the vertical bars in the height profile.

The measured average flake thickness after 10 passes was in the range of 10 nm to 20nm. A selection of AFM images and the corresponding height profiles is shown in figure C.1 to C.21.

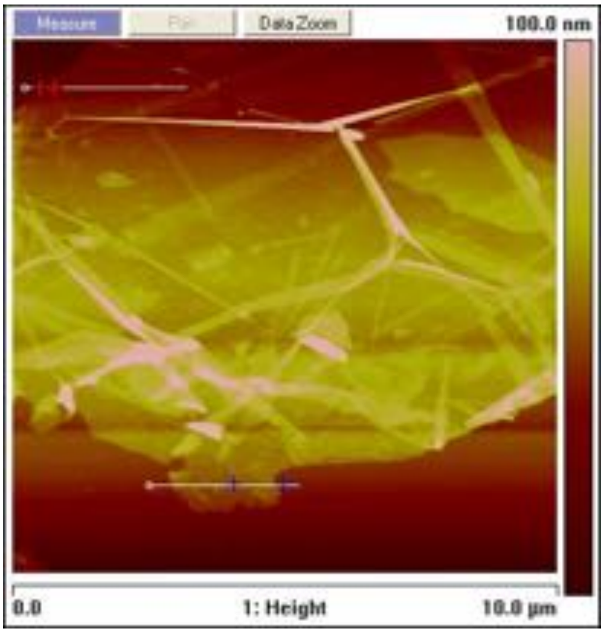

Figure C.1: Height of flake from AFM measurement. The scanned area was 10 µm x 10 µm.

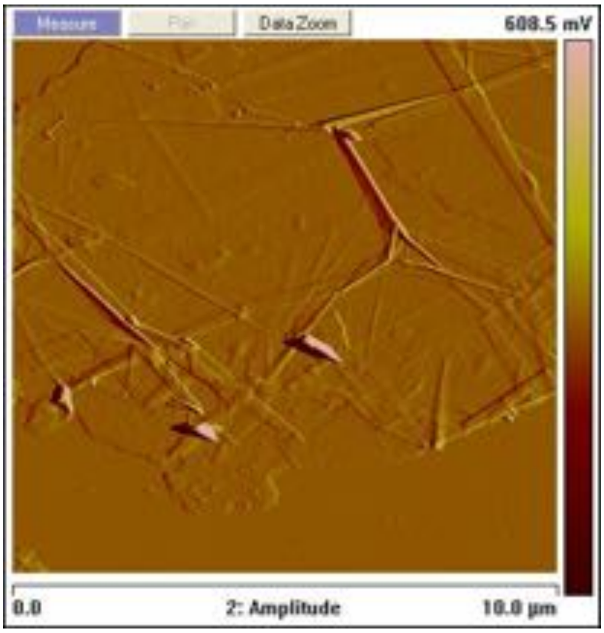

Figure C.2: Amplitude of flake from AFM measurement. The scanned area was 10 µm x 10 µm.

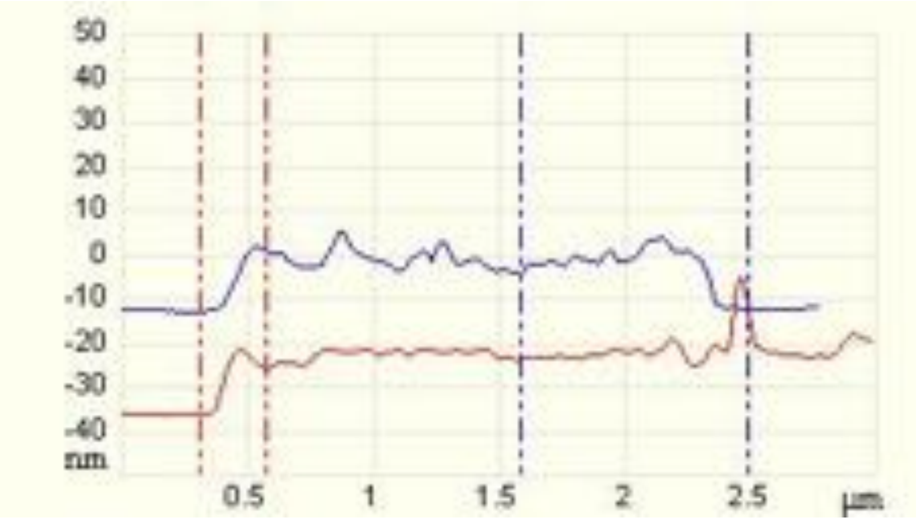

Figure C.3: Height profile from the flake in fig. C.1 and C.2. The height difference (thickness) between the two blue indicators was 7.72nm and 10.57nm between the two red indicators.

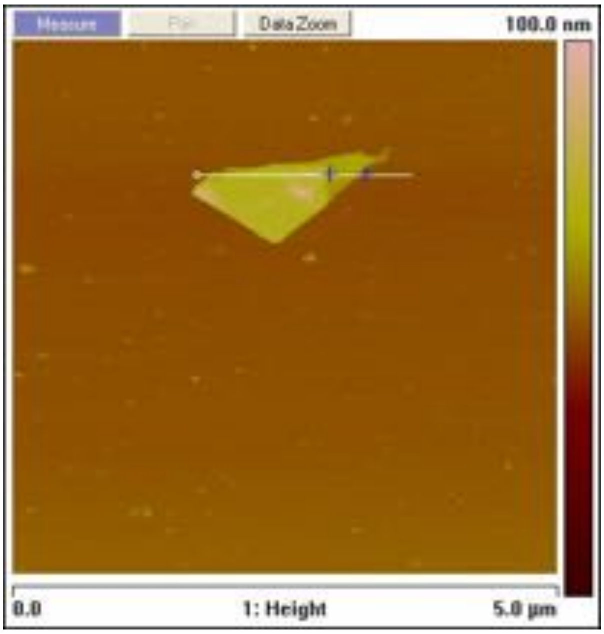

Figure C.4: Height of flake from AFM measurement. The scanned area was 5 µm x 5 µm.

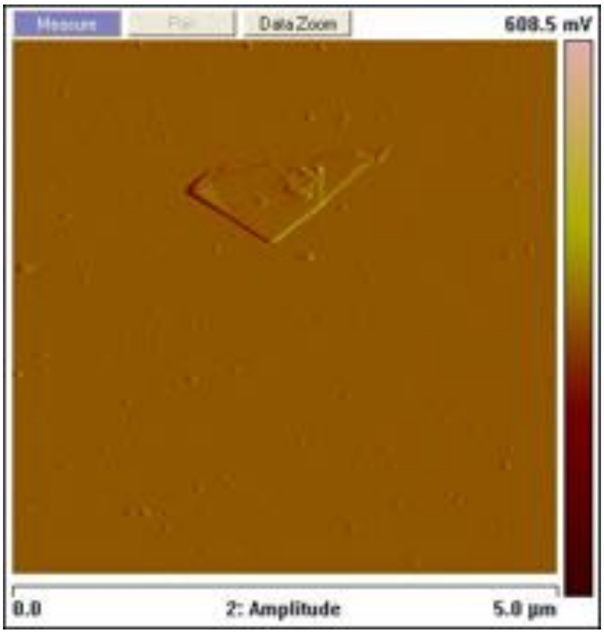

Figure C.5: Amplitude of flake from AFM measurement. The scanned area was 5µm x 5 µm.

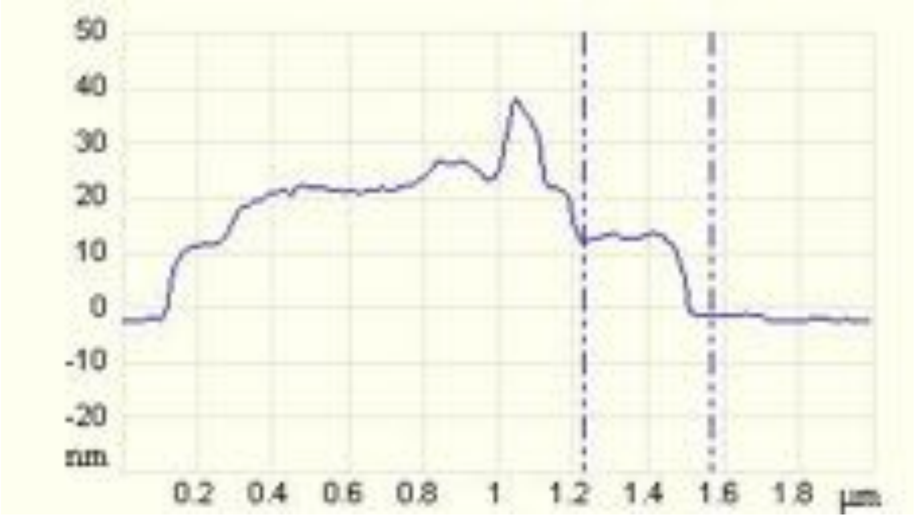

Figure C.6: Height profile from the flake in fig. C.4 and C.5. The height difference (thickness) between the two blue indicators was 13.73nm.

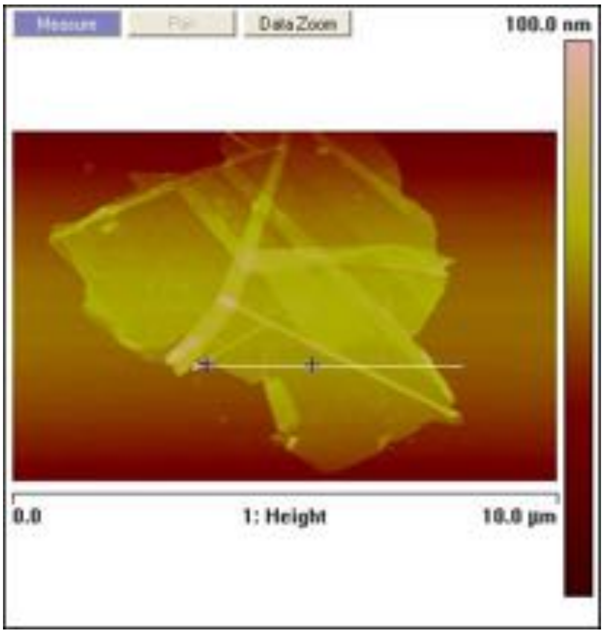

Figure C.7: Height of flake from AFM measurement. The scanned area was 10 µm x 8 µm.

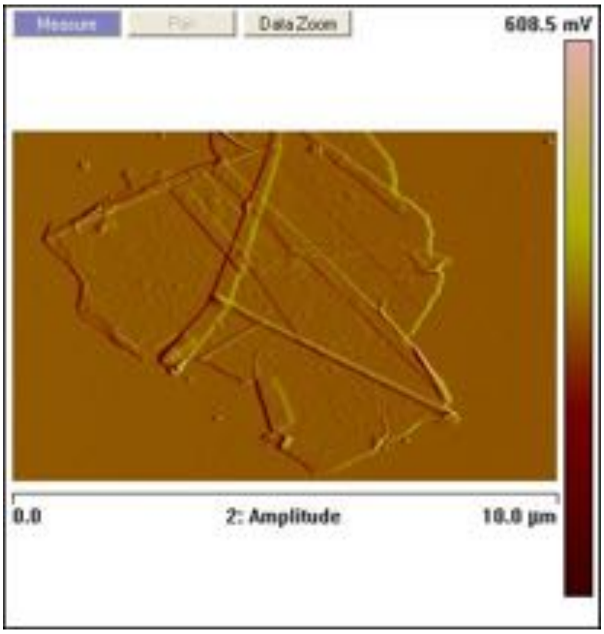

Figure C.8: Amplitude of flake from AFM measurement. The scanned area was 10 µm x 8 µm.

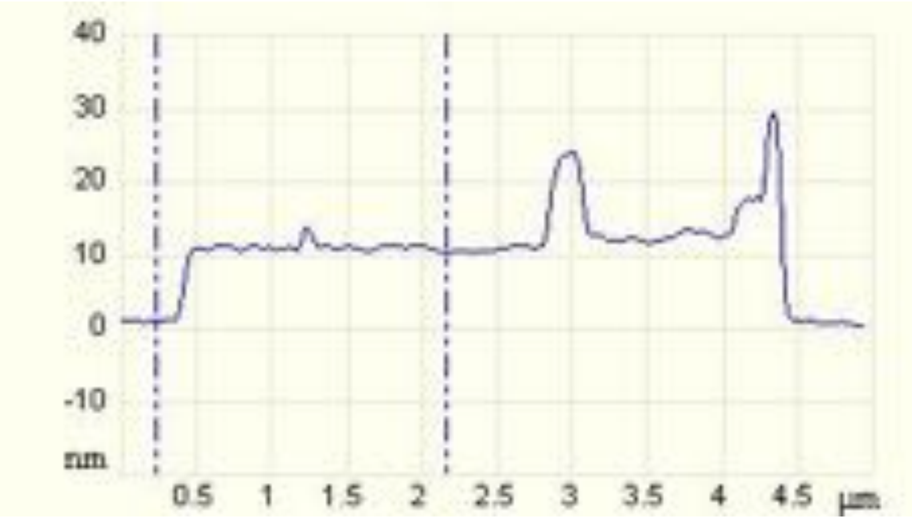

Figure C.9: Height profile from the flake in fig. C.7 and C.8. The height difference (thickness) between the two blue indicators was 9.12 nm.

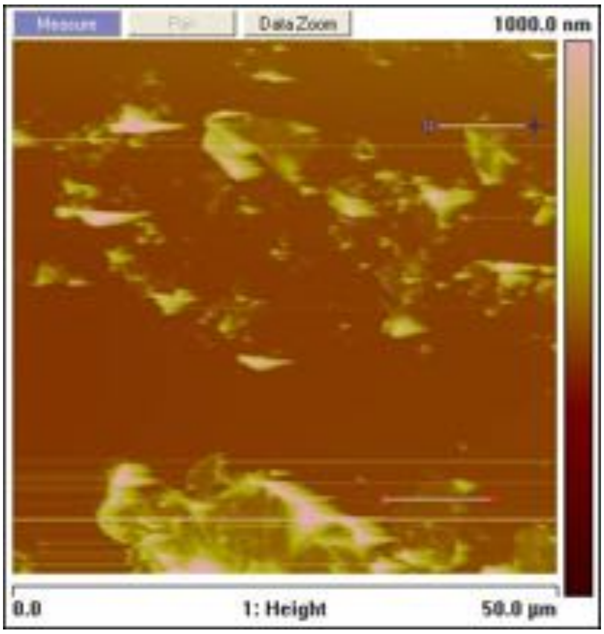

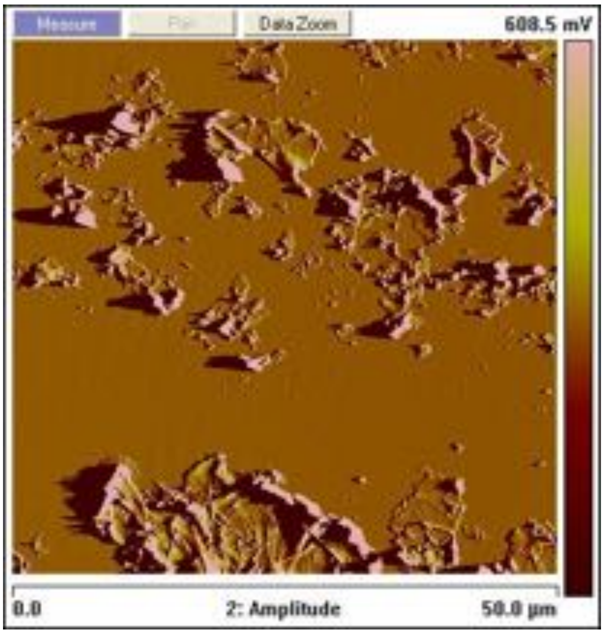

Figure C.10: Height of flakes from AFM measurement. The scanned area was 50 µm x 50 µm.

Figure C.11: Amplitude of flakes from AFM measurement. The scanned area was 50 µm x 50 µm.

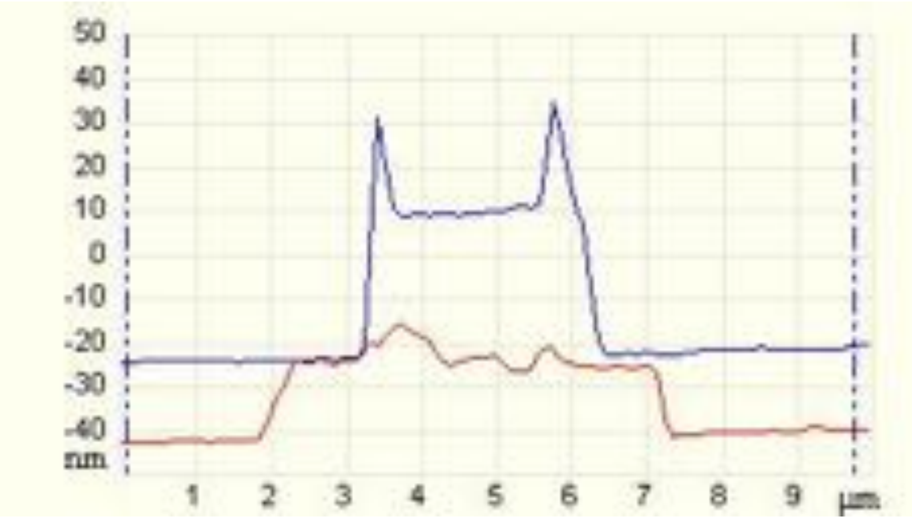

Figure C.12: Height profile from the flakes in fig. C.10 and C.11.

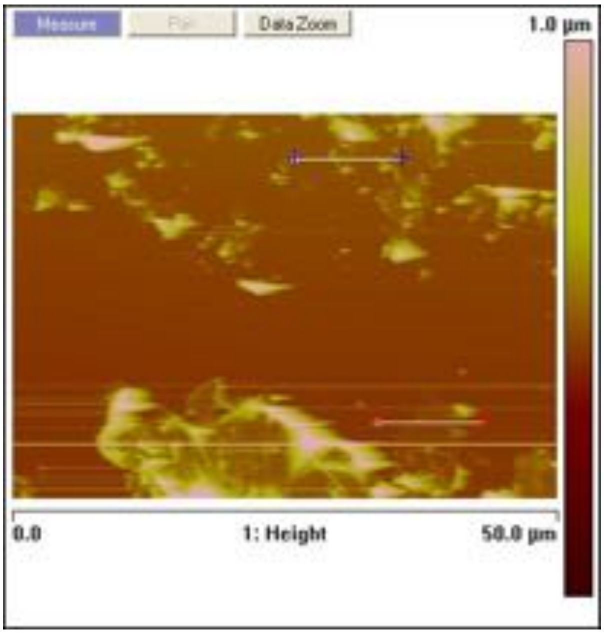

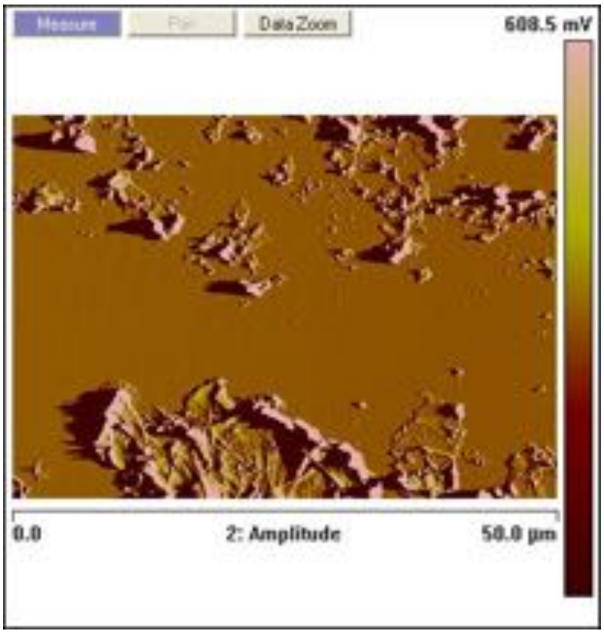

Figure C.13: Height of flakes from AFM measurement. The scanned area was 50 µm x 40 µm.

Figure C.14: Amplitude of flakes from AFM measurement. The scanned area was 50 µm x 40 µm.

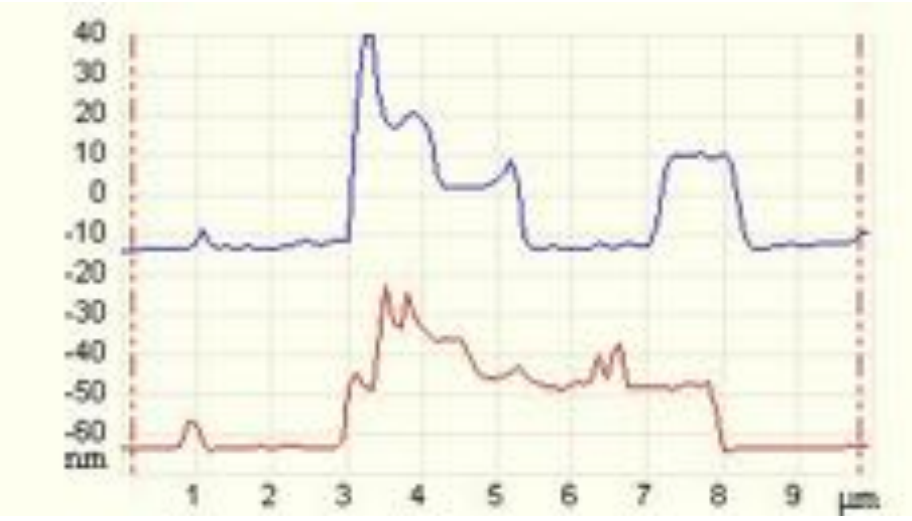

Figure C.15: Height profile from the flakes in fig. C.13 and C.14.

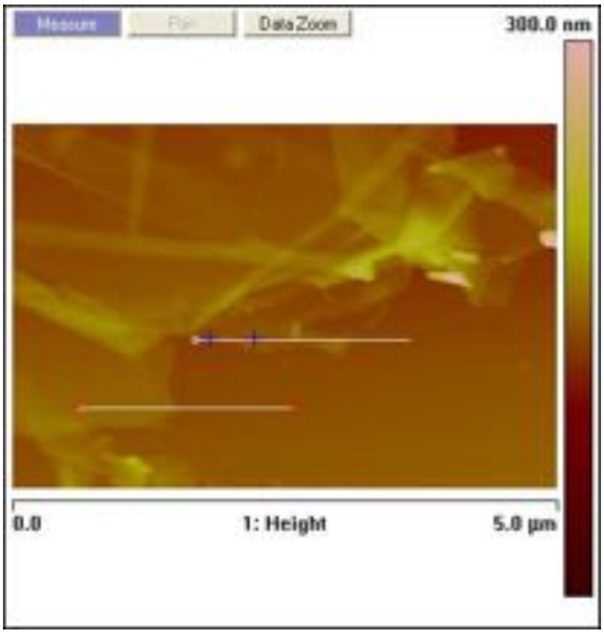

Figure C.16: Height of flake from AFM measurement. The scanned area was 5 µm x 4 µm.

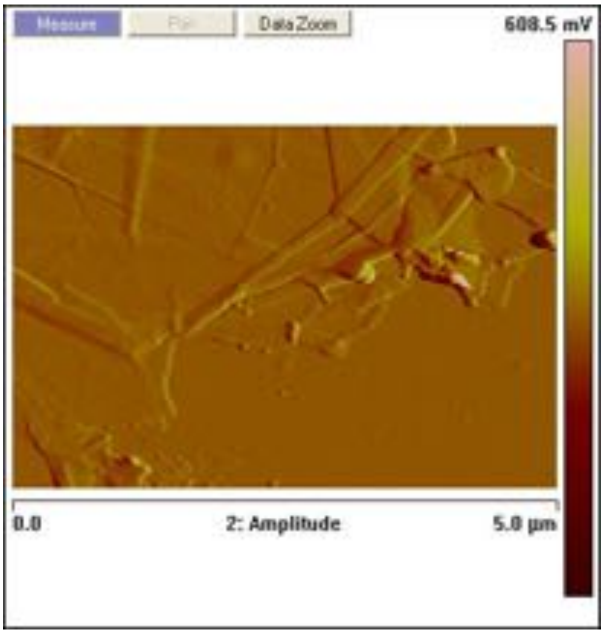

Figure C.17: Amplitude of flake from AFM measurement. The scanned area was 5 µm x 4 µm.

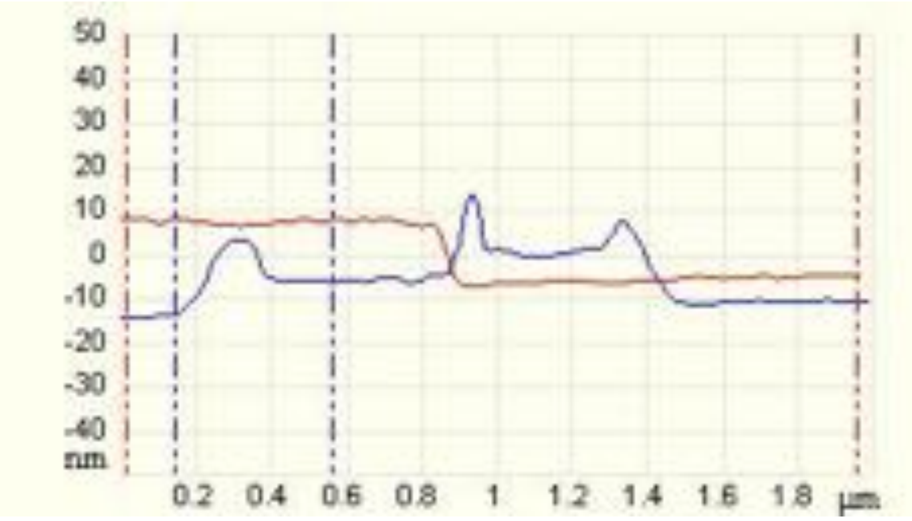

Figure C.18: Height profile from the flake in fig. C.16 and C.17. The height difference (thickness) between the two blue indicators was 7.05 nm and 14.41nm between the two red indicators.

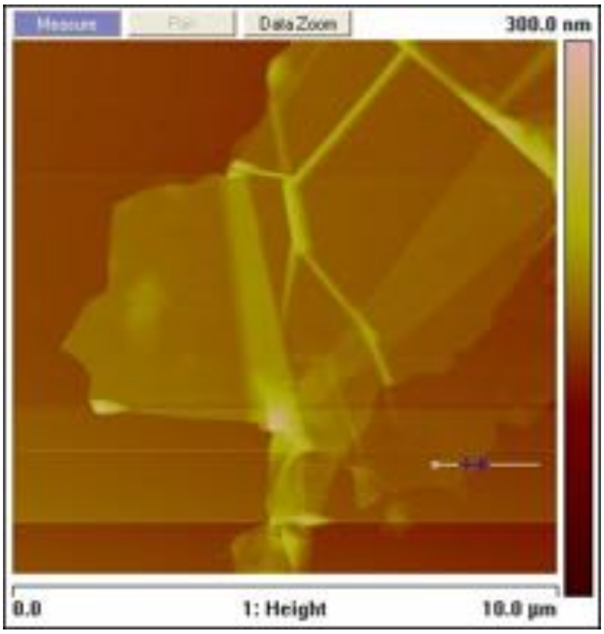

Figure C.19: Height of flake from AFM measurement. The scanned area was 10 µm x 10 µm.

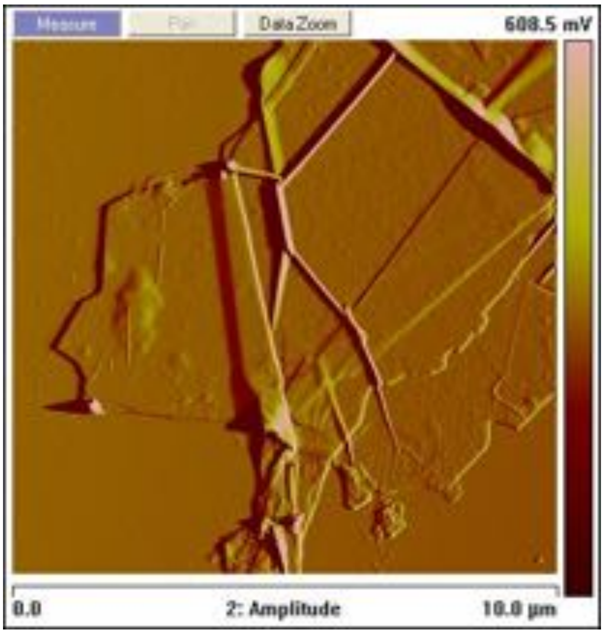

Figure C.20: Amplitude of flake from AFM measurement. The scanned area was 10 µm x 10 µm.

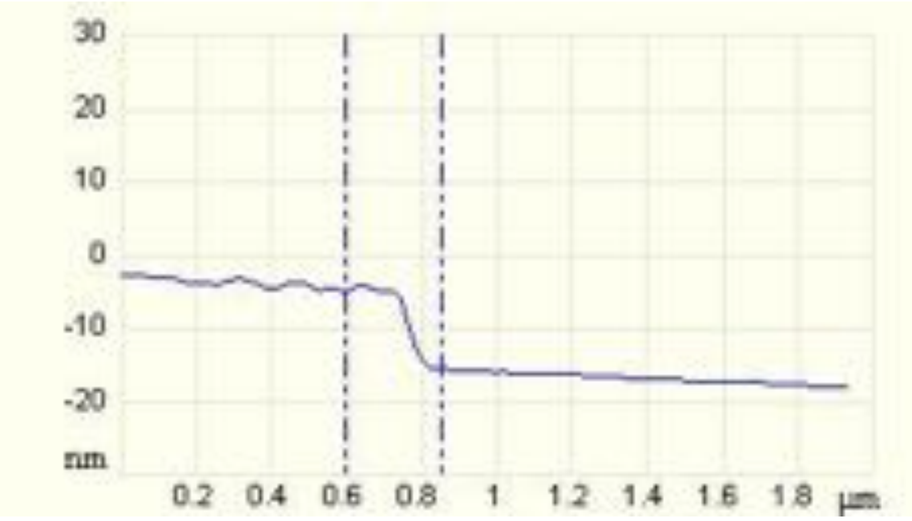

Figure C.21: Height profile from the flake in fig. C.19 and C.20. The height difference (thickness) between the two blue indicators was 10.87 nm.
